# Signed Languages: A Triangular Semiotic Dimension

**DOI:** 10.3389/fpsyg.2021.802911

**Published:** 2022-01-13

**Authors:** Olga Capirci, Chiara Bonsignori, Alessio Di Renzo

**Affiliations:** ^1^Institute of Cognitive Sciences and Technologies (ISTC), National Research Council (CNR) of Italy, Rome, Italy; ^2^Department of Letters and Modern Cultures, Sapienza University of Rome, Rome, Italy

**Keywords:** iconicity, indexicality, simbolicity, semiotics, depiction, signed languages

## Abstract

Since the beginning of signed language research, the linguistic units have been divided into conventional, standard and fixed signs, all of which were considered as the core of the language, and iconic and productive signs, put at the edge of language. In the present paper, we will review different models proposed by signed language researchers over the years to describe the signed lexicon, showing how to overcome the hierarchical division between standard and productive lexicon. Drawing from the semiotic insights of Peirce we proposed to look at signs as a triadic construction built on symbolic, iconic, and indexical features. In our model, the different iconic, symbolic, and indexical features of signs are seen as the three sides of the same triangle, detectable in the single linguistic sign (Capirci, 2018; Puupponen, 2019). The key aspect is that the dominance of the feature will determine the different use of the linguistic unit, as we will show with examples from different discourse types (narratives, conference talks, poems, a theater monolog).

## Introduction

In 1960, William Stokoe published *Sign Language Structure* and showed that signed languages have structural properties comparable to those of spoken languages. Signed languages were analyzed as true languages for the first time, but all the effort was placed in stressing the similarity with spoken languages, minimizing all the features that make signed languages unique, such as simultaneity and iconicity. Signed language linguistics faced the challenge of describing a visual language with instruments provided by the descriptions of a spoken one, forcing American Sign Language (ASL) and other signed languages into molds created for written Indo-European languages.

Throughout most of the twentieth century, different models proposed to describe signed languages were based on a hierarchy: only the lexical units (i.e., standardized in form and meaning signs) were considered at the core of the language, while the “productive signs” (i.e., iconic constructions) were pushed to the linguistic borderline, closer to the level of gesticulation and mime.

In this paper, we will briefly review the history of signed language studies looking for the implication of the language/gesture hierarchy. We will then address how different approaches have tried to overcome this hierarchy, beginning with Christian Cuxac’s semiological approach. Cuxac proposed to describe signed language starting from iconicity, seeing lexical and productive signs emerging from the same iconic and symbolic process. Based on the difference between things and processes pointed out by Langacker (1987), Cuxac distinguished between lexical units and transfer units proposing that a signer can “tell by showing” driven by an illustrative intent (Cuxac, 1999, 2000; Cuxac and Antinoro Pizzuto, 2010).

Ferrara and Halvorsen (2017) and Ferrara and Hodge (2018) applied to signed languages Clark’s theory on spoken communication (Clark, 1996): speakers use their voice and body to communicate employing description, indexicality, and depiction. This theory is based in turn upon the foundational principles of categorization of semiotic signs into symbols, indices, and icons first proposed by Peirce (1994). Ferrara and Halvorsen (2017) and Ferrara and Hodge (2018) proposed a model where the attention is placed on the multiple semiotic modes of expression, and that there are very different ways to display signs according to the signer’s intentions. The authors state that each way is fundamentally different from the other and those signers can use them alone or in combination.

Following a cognitive linguistic approach, Wilcox and Occhino (2016); Occhino and Wilcox (2017); Lepic and Occhino (2018); and Wilcox and Martínez (2020) proposed to overcome the supposed division between lexical signs and gestural elements in sign constructions by building a model based on assumptions from cognitive grammar and construction morphology.

In the present manuscript, we proposed going even further, stating that all these modulations are expressed on a continuum that cannot be broken up into discrete categories, nor is it possible to draw a clear border between lexical and productive signs. There are no pure icons, pure indexes, and pure symbols; that is to say, there are no boxes to categorize a specific type of sign, but rather each linguistic sign can assume all these features. Indeed, a sign will simply show a predominance of either iconic, indexical, or symbolic features, according to the context, the use, or the signer’s intention.

Consequently, the signed language lexicon cannot be divided into symbolic/lexical units and iconic/productive units, tracing the border on the degree of conventionalization of the unit, as the transfer units are also highly conventional constructions. In our model, we consider indexicality, iconicity, and symbolicity as features or semiotic grounds rather than categories, and we, therefore, see them as three sides of the same triangle (Capirci, 2018). The key aspect is the feature’s dominance, represented by the different proportions in the lengths of the triangle sides, with the predominant feature having the longer side. The model will therefore involve equilateral triangles or different scalene and isosceles triangles, depending on the predominance of each feature determining the length of its side.

We will evaluate the effectiveness of this model by applying it to the description of signs found in the following different language uses of Italian Sign Language (LIS): narratives, conference talks, poems and a theater monolog.

## At the Foundation of Signed Language Studies: William Stokoe

Signed languages were long ignored by linguists, being considered a minor form of gestural communication similar to pantomime. In 1960, linguists still considered true language to be only speech, and as such characterized by the vocal-auditory channel, by arbitrariness and discreetness. In 1958, the linguist Charles F. Hockett, 1960 first proposed a list of key properties of language, then developed in his 1960 paper “The Origin of Speech” as the 13 design features. Hockett (1960) writes, “There is solid empirical justification for the belief that all the languages of the world share every one of these features” (1960, p. 90). The very first design feature he discusses, one he feels is “perhaps the most obvious,” is “the vocal-auditory channel.”

However, in the same year, William Stokoe proved Hockett to be wrong about this first design feature, showing that the vocal-auditory channel is not necessary for the development of human language.

“When Stokoe’s monograph was published in 1960, the message it sent was indeed radical. The signs of the deaf, he claimed, were structured, systematic, analyzable as a human language. A revolutionary idea, indeed, that language, human language, could be in sign” (Mcburney, 2001, p.177).

As reported in Stokoe’s biography (Maher, 1996), the intuition that it was possible to study the communication of deaf people through the tools of formal linguistics led Stokoe to propose to the Gallaudet College administration an ambitious research project, the first evidence of which can be found in a 1957 report «…structural linguistic analysis of the language of signs to see if signed languages can be studied as other languages are with a descriptive grammar and lexicon.» (Maher, 1996, pp. 56-7). The need to identify the structural properties of language, in other words, the discrete units that build the linguistic system, is at the very foundation of signed language studies.

It is well known that Stokoe was the first one to identify a cherology and to break up the sign into three formational parameters: handshape, movement, and location. However, in adopting the methods of structural linguistics, Stokoe acknowledged that he had to face the simultaneous nature of the signs when the unitary act of the sign was analyzed in sublexical units by isolating a single point of observation from time to time, an “aspect.” As highlighted by the author in later works (Stokoe, 1980; Armstrong et al., 1995) the phonological analysis takes place on an ideal level “by an act of imagination” (Stokoe, 1980, p. 369)^1^. The aim to describe the lexicon and to provide the first dictionary of ASL led Stokoe to develop an annotation system, now known as Stokoe notation, as a tool for the analysis.

Before the publication of the 1960 monograph, there was no means of writing or transcribing the signs, except for the pioneering attempt of Bébian (1825). Setting aside the challenges of representing visual gestures on manuscript, it was believed that signs were unanalyzable wholes, devoid of any internal structure. Individual signs were cataloged in dictionaries by photographs or drawings, often accompanied by their written language descriptions, as in the first documentations of French Sign Language (LSF) by Sicard (1808) and Laveau (1868). As pointed out by Mcburney (2001), there is a symbiotic relationship between transcription and linguistic analysis, which is acknowledged by Stokoe himself: “the invention of a symbol system for the transcription of the sign language has had to go hand in hand with the analysis of the structure” (Stokoe, 1960, p.30).

Critically, the notation systems used, failed to represent all the signed language units (as, for example, the non-manual ones) and linearly transcribed them, ignoring the simultaneous nature of sign articulation. On the other hand, the widespread use that has been made of “glosses” (”translating” the signs with words of vocal languages) presents a concrete and serious risk of inappropriate segmentation; inappropriate labeling; inappropriate analysis and description of signed structures; “transferring” characteristics of the words to the signs (Antinoro Pizzuto et al., 2010; Antinoro Pizzuto et al., 2008; Antinoro Pizzuto and Garcia, in press).

Since Stokoe’s groundbreaking work, signed languages started to be seen as true languages, but all the effort was put into stressing their similarity with vocal languages (i.e., an “assimilation” to vocal languages). Signed language linguists have tried to reach this goal by forcing ASL and other signed languages into molds that were made for the description of spoken languages—generally, English (Slobin, 2008). Although some early scholars such as Schlesinger and Namir (1978); Klima and Bellugi (1979); Karlsson et al. (1984); Volterra (1987) paid attention to aspects such as the use of space, iconicity, and simultaneity, quite soon the “assimilationist model” became the dominant one, and characteristics that make signed languages unique were often ignored, minimized. Signed languages were characterized by adopting vocal language tools, vocal language based linguistic theory (from a written language perspective), categories, terminology, and analyses.

## Iconicity as a Semiotic Engine: Christian Cuxac

A new linguistic model was first proposed by Cuxac in 1985 attributing to iconicity a crucial, formal role in shaping signed languages discourse and grammar (Cuxac, 1985, 1999, 2000, 2004, 2013). According to Cuxac, all signed languages are grounded upon the basic capacity that signers have in iconicizing their perceptual/practical experience of the physical world and make a structured use of the shared physical-linguistic space of signed discourse.

Cuxac’s research starts from a reflection initiated by Jouison in the late 1970s that was made public with a collection of his writings in 1995. Jouison soon rejected the chereology initially proposed by Stokoe, insisting on the fundamental role of the whole body in the signed discourse and on iconicity. Jouison emphasizes that the mimetic aspects of signs in no way detract from their being linguistic acts, and focuses primarily on iconic discursive structures, from which Cuxac’s linguistic reflection will start.

According to Cuxac (1999, 2000) signed languages exploit the signer power to iconizing their perceptual and bodily experiences. Iconization does not reside only in the formation of the sign, at the origin of its etymology, but remains a source of creativity at the synchronic level, which the signers can draw on to structure their discourse driven by an illustrative intent.

Cuxac (1985, 1996, 2000) proposed a semiotic model in an enunciative approach for the analyses of signed discourse. This model is in line with the following works undertaken by the French research group (Garcia, 2010; Fusellier-Souza, 2006, 2012; Garcia and Sallandre, 2020) and by the Italian research group (Pizzuto and Corazza, 2000; Russo Cardona and Volterra, 2007; Volterra et al., 2019). According to these scholars, in contrast with the structuralist perspective to approach the linguistics of the signed languages, it was necessary to abandon influences and preconceptions coming from linguistics of spoken languages, especially the generativists’ approach.

Cuxac argued that it is necessary to start from the internal regularity of the language to study it, without projecting analytic categories from linguistics of vocal languages. The author was inspired, among others, by the pioneering work of Jouison (1995), who draw attention to the bodily components (especially eye gaze and body movements) and to the linguistic analysis of iconic construction in LSF. Cuxac shed more light on the incidence of iconicity in grammatical structures, elaborating the model of the Highly Iconic Structures. The notion of a structure that is built on iconicity was precisely aimed at recognizing the linguistic value of iconicity as a grammatical structure of the signed languages: “These constructions are verbal (that is, linguistic) precisely because they are based on structures, that is, they are composed of constrained elements that fit into paradigms” (Garcia and Sallandre, 2020, p. 5).

Cuxac believed that the grammatical classes of signed discourse differ significantly from those used for vocal languages (Beccaria, 1994; Simone, 2008) where they are called verbs, nouns, adjectives, and conjunctions. According to the theoretical framework of cognitive grammar (Langacker, 1987), grammatical classes could be distinguished instead in things and processes, categories grounded in our cognition. Cuxac (1999) found this perspective well suited to describe LSF and later applied it to other signed languages as well. In fact, he studied different types of iconicity in the signed discourses led by different iconic/visual and lived experiences. In this way he found, identified, and distinguished different units of meaning in the signed discourses: standard signs (standard lexicon) and Highly Iconic Structures (HIS), now called lexical units (UL) and transfer structures, that generate a multitude of transfer units (UT).

“This model was progressively developed from the early 1980s on the basis of close, frame-by-frame, analysis of long spontaneous discourse corpora, recorded *in situ* (Cuxac, 1985, 1993, 1999). The methodological decision to work on corpora, setting out from a functional and therefore semantically centered perspective (a top-down approach), was unique at the time (and remained so until the 1990s), as research on other signed languages had long been focused primarily on elicited data such as decontextualized sentences” (Garcia and Sallandre, 2020, p. 5).

One of the effects of this iconization process is to endow signed languages with an additional semiotic dimension compared to vocal languages. In signed languages, there are two ways of signifying (Cuxac, 2000; Russo Cardona and Volterra, 2007; Cuxac and Antinoro Pizzuto, 2010; Volterra et al., 2019): (1) by “telling without showing” - using: (a) units that are broadly comparable to vocal languages’ content words, which we will call here “lexemic units” (LU); (b) pointing signs realized manually but also visually, by re-directing the signer’s gaze in the signing space -; (2) by “telling and showing,” thereby producing complex structures that can be characterized as “transfer units” (TU) and are unique of the signed modality. A most relevant feature of TU is that they can be combined among themselves, or with LU, to encode information on two (or even more) referents in a multilinear, simultaneous fashion that has no parallel in speech. Gaze patterns play a key role in distinguishing LU from TU. When producing the LU the signer’s gaze is oriented toward the addressee. In contrast, when producing TU the signer’s gaze is away from the addressee and their head and body posture clearly differ from those used in producing the LU.

## New Non-Structuralist Approaches

The approach initiated by Cuxac and now known as the Semiological Approach (e.g., Cuxac, 1999; Fusellier-Souza, 2006, 2012; Sallandre, 2006; Cuxac and Sallandre, 2007; Garcia, 2010; Garcia and Sallandre, 2020) remained little known for a long time and still does not have a great resonance among modern-day signed language researchers. Linguistic research on signed languages continued trying to respond to two pressing practical priorities: the need to fix citation forms of signs for new dictionaries and the need to have notation systems for annotating corpora.

Throughout most of the twentieth century, linguists were busy constructing models in which discrete elements belong to discrete categories, and in which various types of rules combine those categories of elements to produce words, phrases, clauses, and sentences. In this attempt, different functionalist and cognitive approaches to signed languages tried to develop models opposing the structuralist view. Nevertheless, the need to create dictionaries and corpora pushed signed language research to continue to set clear boundaries between what was described as fully lexicalized signs, namely those which could easily enter in a dictionary. They have fixed form and meaning, as well as the transfer units, seen as more gradient and therefore gestural.

### Signs at the Core and Signs at the Edge of Language

One of the first models of this kind was proposed by Johnston and Schembri (1999) and later revised by Cormier et al. (2013) in a simplified version (2013).

Johnston and Schembri’s (1999) proposal is motivated by the practical need to establish which linguistic units in Auslan (Australian Sign Language) are best entered in a dictionary and which are best treated in grammar: “In the first instance, one needs to discriminate between non-linguistic visual-gestural acts (gesticulation, gesture, and mime) and linguistic visual-gestural acts (signs). The lexicographer is concerned with the latter” (Johnston and Schembri, 1999, p.115). Following this assumption, a distinction is made between “lexicalized” signs and those which are partly lexical or non-lexical.

Fully lexical signs are those defined as conventional in their form and meaning: “A lexeme in Auslan is defined as a sign that has a clearly identifiable and replicable citation form regularly and strongly associated with a meaning which is (a) unpredictable and/or somewhat more specific than the sign’s componential meaning potential, even when cited out of context, and/or (b) quite unrelated to its componential meaning potential (i.e., lexemes may have arbitrary links between form and meaning)” (Johnston and Schembri, 1999, p. 126).

This “frozen” lexicon, in line with Brennan (1990), “is a list of stable forms and stable meanings (i.e., the lexemes) which is known only to a user of any particular sign language” (Johnston and Schembri, 1999, p. 131).

Johnston and Schembri thereby build a gestural hierarchy and sign typology, visually represented in an image of concentric circles in which the signs at the center, the core, are fully linguistic, while those encompassing circles are less and less linguistic, with the non-linguistic gestural forms being in the outermost circle.

The core is defined by characteristics such as full arbitrariness between form and meaning, conventionality, non-componentiality, and the stability of forms and meanings. What is “relegated” to the periphery as partially lexical or non-lexical are “Signs (lexemes) which show no obvious form/meaning relationship” (Johnston and Schembri, 1999, p. 131). The complex signs, characterized as partly lexical, have properties of gradation, while the non-lexical signs are unconventional bodily actions that show meaning, and are dependent upon context for their interpretation (Whynot, 2016).

However, the division between a “frozen” core and a “productive” edge of the lexicon can be questioned by considering if, on the contrary, the special features of linguistic signs (vocal or signed) are precisely their being “productive” and therefore unstable, vague in meaning, modifiable by speakers, iconic, compositional.

This hierarchical model proposed by Johnston and Schembri [inspired by Liddell (1995)], has been adopted by others in several subsequent works, albeit with some variations. Whilst not altering its substance they have tried to divide the components of signed languages into “core lexicon” completely linguistic, and increasingly less “linguistic” peripheries which slope outward toward the limit of the gestural or non-linguistic.

Depicting signs were regarded as both linguistic and gestural elements (Schembri, 2001; Liddell, 2003; and Schembri et al., 2005). Pointing signs have been characterized as hybrid (partly conventional, partly non-conventional) forms, and it has been suggested that points are gestural, much like co-speech gestural pointing that occurs in spoken languages (Johnston, 2013).

Johnston and Schembri (2010) propose a Table (p. 27) in which they present the linguistic universe of signed languages divided into various categorizations identifying two major types of signs in signed languages with the different names having been given to them by different authors (e.g., Frishberg, 1975; Liddell, 1977; Supalla, 1978; Liddell and Johnson, 1986): fully lexical signs (regular signs, frozen signs) and partly lexical signs (productive signs, non-lexical signs, depicting signs).

Cuxac (2000) has also been included in this “binary” vision, focusing on “standard sign” vs. highly iconic structures, but in a somewhat incorrect way, as in Cuxac’s view, these categories are in no way comparable to the vision here expressed by Johnston and Schembri. According to Cuxac, transfers cannot in any way be confused with pantomimic forms since they are based on a real linguistic structure which is an alternative to the standard lexicon; conventionality is always present. As Cuxac and Sallandre (2007) clearly point out: “among several coexisting forms of iconicity in LSF, even the most imagistic of them are organized in macrostructures on an initial level, making short work of the equation “iconic” means “unstructured” (Cuxac and Sallandre, 2007, p. 14); “Linguistically speaking, iconicity poses no theoretical problem for these structures, since the intent is deliberate. Wondering why this type of iconicity exists is as irrelevant as asking why a figurative painter will paint naturalistic subjects. The interesting question is how. With these different examples, we hope to have shown that structures and iconicity can go together” (*ibid*. p. 20).

We have said that this hierarchical approach for Johnston and Schembri (1999) was motivated by the need to establish which linguistic units in Auslan are best entered in a dictionary. Now let’s see what Cormier’s needs were.

Cormier et al. (2012) aim to code or annotate natural signed language data and therefore for these authors identifying the lexical signs has fundamental implications for the analysis: “there is nearly always a need to identify tokens within the signing stream which are lexical signs (in the sense of the core lexicon) versus those which are not” (*ibid.* p. 344).

These researchers were particularly interested in coding constructed actions (CAs) and proposed to evaluate the degree of gestural component of each type of construction according to the following consideration: “Cues for gestural status of handling/embodiment could be the overtness of constructed action used (as marked by the number of articulators used and/or degree to which the various articulators are active…), or the degree of iconicity between production and referent such that the more overt the constructed action and/or the higher the iconicity between production and referent, the stronger the character viewpoint gestural status” (*ibid.* p. 344).

In this reasoning, it seems that what underlies the distinction between linguistic and gestural is the presence of the so-called non-manual parameters, iconicity, and simultaneity (the use of several articulators at the same time). Therefore, this approach seems to suggest that the non-manual components, iconicity, and simultaneity are paralinguistic or gestural properties.

Cormier et al. (2013) proposes a simplified version of the Johnston and Schembri model (p. 373) with three concentric circles: at the “core” the standard signs (lexemes); then the productive signs that include depicting constructions (DCs) such as whole entity constructions (“non-core lexicon,” Brentari and Padden, 2001); and finally to the extreme periphery the “gestures and mime,” non-lexical means “*via* CA, to portray actions of referents by full or partial mapping of articulators onto actual (or perceived) actions, thoughts, utterances, or feelings” (*ibid.* p. 373).

Surely these models with concentric circles can have their usefulness for selecting the signs to be included in a Dictionary, or to annotate the corpora with identifying glosses (ID-glosses, e.g., Crasborn and Sloetjes, 2008; Johnston, 2008, 2014; Cormier et al., 2015), but are we sure that these hierarchical models that attribute values of “linguisticity” reflect the nature of signed languages (and languages in general)? Are we sure that everything that is iconic, variable according to the context, “corporeal,” simultaneous, is not also conventional and arbitrary?

These scholars undoubtedly have the merit of having opened and widened the field to the study of these special aspects of signed languages, - starting for example from the impressive analysis and consideration of the non-manual components that Cormier makes in her study of CAs - but why give them a “non-linguistic” status?

Cuxac was the first to begin to investigate these highly iconic structures or transfer units and to make a division between a “telling” mode and a “telling by showing” mode. However, it must be acknowledged that he has never placed these two levels in a hierarchical way nor has he ever considered “showing” as a non-linguistic or “gestural” semiotic plane. Indeed, he has used the term “structures” precisely to underline the systematic nature of the iconic plane of “showing.”

Nevertheless, over the years until today, Cuxac and the Semiological Approach continue to be little known and cited, while many of the new “models” proposed to describe signed languages seem to have internalized (almost like a dogma) this general view of language in which there is something more linguistic than the other.

### Different Modes of Expression for Different Types of Signs

Hodge and Johnston (2014) make it clear that they belong to the broadly cognitive-functional construction grammar perspective. In their adoption of a perspective, that we could call *cuxachian*, they declare that signers, as well as speakers, construct their meaning using “semiotic signs of different types” and these different semiotic modes are those of telling and showing. Thanks also to the development and availability of time-aligned multimodal annotation software like ELAN that allowed building multimodal vocal languages and signed languages corpora (e.g., Crasborn and Sloetjes, 2008), according to the authors it is possible to investigate and “count” the prevalence in the signing of these two semiotic resources.

So far, the model appears as a more recent version of Cuxac’s theory, but again the division emerges between a linguistic level, telling, and less than little or not at all linguistic or gestural level, showing. The authors state that while “formal and theoretical linguists have typically focused on describing how speakers and signers “tell” meaning (…) More recently, this focus has evolved to also consider how language users manipulate various semiotic resources to visually represent and “show” meaning to prompt conceptualizations for their interactants” (Hodge and Johnston, 2014, p. 265).

The realm of showing includes iconicity, use of non-manual components, and simultaneity. But once again the authors cannot avoid providing a hierarchy to these worlds and hierarchize between fully lexical, partly lexical, and non-lexical/gestural: “Signs vary gradiently from fully lexical, through partly lexical, to non-lexical according to degrees of conventionality, complexity and schematicity.” (*ibid.* p. 267). They also specify that “Partly lexical signs have only some characteristics specified in their form (typically handshape and orientation); all other specification emerges from mapping these forms onto the signing space.” And that “Pointing signs (also known as pronouns and indexing signs in the SL literature) and depicting signs (also known as classifier and polycomponential signs) are two major sub-classes of partly lexical signs” (*ibid.* p. 267). While defining the “non-lexical signs, as “singular events” during which interactants enchronically interpret a form as “standing for” a meaning (Kockelman, 2005)” (*ibid.* p. 268). This category includes (again) CAs and DCs.

This hierarchical model, although starting from a different approach (cognitive linguistic), ends up resembling (too much) the approach of the formal and theoretical linguists, as it is presented, for example in the target article by Goldin-Meadow and Brentari (2017). At least some residues of structuralism are therefore shown. Any sort of expression in signing that cannot be analyzed in discrete, categorial terms is defined as gestural. As discussed before, this approach entails the risk of framing as language only a really small portion of signed languages, while excluding and relegating to the darkness of non-linguistic and gestural domain what does not fall into this category. That is, all the non-manual components, the transfer units (TU) or constructed action (CAs), depicting constructions (DCs), and considered as co-sign gestures. Again, a universe divided into two blocks, black and white, linguistic and non-linguistic, within rigidly closed, and separate categories.

Finally, we come to the model proposed by Ferrara and Hodge (2018; see also Hodge et al., 2019). Ferrara and Hodge proposed a theory of language built on Clark’s (1996) theory of language use as “actioned” *via* three methods of signaling: describing, indicating, and depicting. This theory is in turn based upon the foundational principles of symbols, indices, and icons first proposed by Peirce (1955).

Ferrara and Hodge (2018) state that: “each method is fundamentally different from the other, and they can be used alone or in combination with others” (p. 1). Subsequently, they define the three types of signs in a very rigid way. For example, they say that symbols - the category in which they include the lexicalized manual signs of signed languages - are signaled through acts of description. Afterward, they refer to the Dingemanse definition of descriptions as “typically arbitrary, without a motivated link between form and meaning … these symbols are discrete rather than gradient.” Later, the definition of icons presented as partially depicting meaning trough perceptual resemblance in contrast with symbols… “they are gradient, varying.” This category includes typically “mimetic enactment of people, animals or things” (*ibid.* p. 4; Dingemanse, 2015, pp. 950–951). Icons and depictive signs (or, in other terminology, TU, CAs, and DCS) are considered to be on a par with gestures [a sort of co-sign gestures as in Goldin-Meadow and Brentari (2017)]: “Depicting signs have been compared in varying degrees to the iconic and metaphoric manual gestures (also known as referential gestures) produced as part of spoken language discourse” (Ferrara and Hodge, 2018, p.5).

Even if this approach represents an attempt to overcome the dichotomy between gestural and linguistic elements, it is still possible to see the division between white building blocks (symbol, arbitrary, and categorical) on one side and black building blocks (icon, motivated, and gradient) on the other. In fact, if these building blocks can come together in the signed expression - as the authors correctly points out - why divide them so rigidly? More importantly, is it possible to find a pure symbolic unit in signed language, without any indexicality or iconicity? In other words, is it possible to separate the depicting, describing, and indicating functions in language, or is it rather a matter of dominance of one function over the other?

### New Insight From Cognitive Linguistics: A Continuum Between Fixedness and Schematicity

Armstrong et al. (1995) proposed to look closely to the similarity between gestures and signs, introducing what Janzen (2006) nicely defines the continuous account in signed language research, carried on by cognitive linguistics. This continuous account is used to reflect on grammaticalization and gestures-sign interface in Janzen and Shaffer (2002); Wilcox (2004); Wilcox et al. (2010), suggesting that gestural materials are conventionalized as lexical or grammatical items in signed languages, in a transitional, and not abrupt, manner.

More recently, in their commentary to Goldin-Meadow and Brentari (2017); Occhino and Wilcox (2017) pointed out very clearly that the language versus gesture dichotomy based on discreteness versus gradience is too simplistic. The authors explained how a usage-based framework suggests that networks with different levels of complexity, specificity, and schematicity emerge from language use. Considering this approach, gradient elements are not seen as gestural, but simply as linguistic.

Following this approach Wilcox and Occhino (2016); Martínez and Wilcox (2019); and Wilcox and Martínez (2020) introduced the concept of Place as a symbolic structure largely exploited in signed discourse. A Place is a pairing of a specific meaning and a specific location in the signing space in the context of a usage event, it is used in placing and pointing constructions. For example, if a signer wants to make a comparison, she will probably use a placing construction creating a place for each element, signing the signs in a specific location, for instance to the right and to the left. During the signing discourse, the two Places can be recruited again to refers to these elements, using again a placing construction, or a pointing (Wilcox and Martínez, 2020). This perspective, inspired by Cognitive Grammar, shows how it is possible to explain gradient and not listable signed units within a linguistic framework, without having to resort to a “mixed model” with gestural gradient elements seen along with discrete linguistic units.

Recently, Lepic and Occhino (2018) have discussed the language versus gestures issue starting from the rule/list fallacy proposed by Langacker (1987, 2008). The supposed division between grammar rules and lexicon should be rejected since it is imposed by the linguist’s need to have abstract categories. In contrast, linguistic rules are schema emerging from use. The point is not to deny the existence of regularities, which are in fact undeniable, but rather to see rules and usage as a whole. Linguistic regularities are not independent operations from the matter on which they are applied, but on the contrary, they are schemes or organizational lines that emerge from the linguistic matter itself and from the way in which it is associated. In the same way, signs that have been defined as the lexical units at the core of signed language cannot be completely separated from the so-called productive lexicon, they co-exist with highly iconic properties and schemas, and have a gradient rather than discrete internal structure.

The authors, building on Langacker’s insights, insist on how the theoretical framework of cognitive linguistics can aid research on signed languages by leaving aside the idea of language as a structure of discrete, enumerable elements. Cognitive linguistics embraces an idea of language analyzable in terms of constructions, of conventionalized pairings of form and meaning, both containing holistic or discrete elements and yet organized in a system, conceived as a network. Conventionality has been seen as a foundational property in the human language since phonology, morphology, grammar, and lexicon are described as a continuum of conventional linguistic units (Croft and Cruse, 2004).

Lepic and Occhino (2018) use Construction Morphology to show that both transfer and lexical units do not have to be divided since: “construction-theoretic analysis instead treats entrenched, highly fixed “lexical” signs and more schematic and productive “classifier” signs alike as learned pairings of form and function (or meaning). Rather than assigning individual sign tokens to distinct domains of linguistic knowledge, all sign constructions can be considered primarily meaningful wholes that also exhibit gradient internal structure” (Lepic and Occhino, 2018).

Furthermore, a linguist may look at signs and analyze them without seeking help from the dichotomy between gesture and language: all signs are equally pairs of form and meaning with different levels of fixedness and schematicity. The usage-based approach has been successively employed to explain “lexicalization” in signed language by Lepic (2019), the author shows how there is not a clear distinction between holistic and structural properties in signed constructions, and it is therefore better to set the analysis on degrees of fixedness rather than on categories.

## Beyond Categories: Three Faces of the Same Triangle

The tendency toward categorizing and discontinuity is a product of researchers’ needs (e.g., the need to establish which linguistic units are best entered in a dictionary, Johnston and Schembri, 1999; the need to code or annotate signed language data, Cormier et al., 2012) and originate from an alphabet-based culture (of written languages) which has influenced, even if at times subconsciously, our metalinguistic reflection.

Usage-based approaches have laid bare ever-growing doubts about the correctness of the distinction between lexicalized and productive - partly lexicalized - signs; between symbol, icons, and indexes; between the modes of describing, depicting, and indicating. Ferrara and Halvorsen (2017) stress that signs can be used as both descriptions and depictions and should rather be considered as somewhere on a continuum between these two strategies instead of separating them into two distinct categories. In any case, the need to annotate often pushes us, as linguists, to fall into a categorization trap as if a sign can belong to one or the other “category” in an exclusive way.

To overcome the categorization trap, signed language research can find help from the field of semiotics. In fact, semiotics teaches us that: “The same signs can be icons, indices, or symbols depending on the interpretive process” (Deacon, 1997, p. 72). As is well established, Peirce conceives any act of signification as a triadic phenomenon, concerning the sign, the object, and the interpretant. Each sign represents the object to a certain respect, projecting in the sign some features of the object: ‘‘the sign stands for something, its object. It stands for that object, not in all respects, but in reference to a sort of idea, which I have sometimes called the ground of the representamen’’^2^ (Peirce, CP 2.228).

In this sense, the icon is constructing a relation of resemblance with its object, the index of proximity or a cause/effect relation, a symbol of a conventional relation. Nevertheless, Peirce’s notion of icon, index, and symbol can be interpreted in terms of features instead of fixed distinct categories. The same sign (even in the same context) has all three features in itself, sometimes in equal measure/gradation, other times with a predominance of one over the others. As Kockelman (2005, p. 246) points out “it is best to talk about iconic, indexical, or symbolic grounds, rather than to talk about icons, indices, and symbols *per se*.”

Puupponen (2018) in her Ph.D. thesis (2018) embraces this interpretation and argues that “Because of these inclusive relations in the Peircean theory, Peircian categories are very useful for studying (…) Sign languages that present a variety of iconic phenomena imbued with conventional and arbitrary aspects for which the categories elaborated by the linguistics of vocal languages are not sufficient.” (p. 43).

Also with respect to iconicity, the field of semiotics has a lot to teach to linguistics. Iconicity is often interpreted as the opposite of arbitrariness and conventionality, making an equation (more or less conscious and explicit) between iconicity and naturalness/necessity. In part, this interpretation of iconicity derives from or is explicitly made to depend on a certain interpretation of Saussure, whose notion of arbitrariness leaves instead ample room for forms of iconicity (diagrammatic).

In an informative essay on iconicity and metaphor “The Map laid down upon the island” the Italian linguist Tommaso Russo (2004) offers us a “reading” of Peirce that establishes the non-equivalence of iconicity and naturalness: “Peircian iconicity presupposes that the iconic relationship manifests itself only on the basis of identifying a perspective through which sign and object enter into a relationship. The signs resemble their objects starting from a complex series of habits and conventions to which they are subjected and which govern the semiotic process, in its triadic dimension. This process, in fact, always includes a sign, an object, and an interpretant, therefore a series of clothes.” (p. 47). Each icon, Peirce points out, shows a resemblance to the object under a certain respect; we need to refer to certain implicit conventions and a way of looking at the object represented. Each icon, just as Plato had argued, is based not only on similarity but also on a habit of representing the object in one way rather than another: “the sensorial and qualitative characteristics of the sign sanction iconic relationships only thanks to the mediation of habits and norms that are part of linguistic competence.” (*ibid.* p. 48).

By identifying iconicity and naturalness, one runs the risk of presenting language as if it were merely reflecting characteristics already given in the real world. On the contrary, an iconic sign never mirrors the referents but always mediates a certain meaning through projecting a resemblance. “In languages, the cases in which the iconic dimension and that of arbitrariness and variability coexist and illuminate each other are, indeed, much more relevant and worthy of consideration than those in which these two forces seem to oppose or exclude each other” (*ibid.* p. 52).

How can highly iconic language phenomena coexist with the formal and structural needs of a linguistic system? The coexistence of iconicity and arbitrariness must lie at the heart of the complex interplay between the formal requirements of the linguistic system and the pragmatic constraints which guide the interpretation of a linguistic utterance (Fontana and Volterra, 2020).

The plasticity of linguistic units makes it possible for these to be interpreted in context and change meaning and form (De Mauro, 1982, 2000). In fact, one of the main semiotic features of linguistic signs (signed or spoken) is their indeterminacy, that allows the human language to be inherently plastic. Pietrandrea (in press), has recently shown the relevance to De Mauro’s notion of plasticity for signed language research. As shown by De Mauro (1982, 2000) the plasticity allows the signer or the speaker to negotiate the meaning of a linguistic units, as in the case of technical jargon, or to extend the meaning of a unit to a metalinguistic use. Because of this plasticity, linguistic units do not afford a complete and exhaustive interpretation of an utterance and need some pragmatic prompt for the interpretation to take place. Discursive iconicity is thus a major structural resource of signed languages permeating every level of the language and acting as a major pragmatic constraint in utterance interpretation.

In her book “From Speech to Grammar. Construction and form of spontaneous texts,” Voghera (2017) starts from the perspective of the modality of face to face communication which places the indeterminacy, vagueness and low definition of the sign in the foreground, alongside the elasticity and instability of the spoken texts (see also Fontana et al., 2017; Volterra et al., 2019; Fanelli and Volterra, 2020; Fontana and Volterra, 2020).

“The form takes shape little by little, because the speaker (…) constructs the meaning along the way, also relying on the more or less explicit cooperation on the part of the recipient” (Voghera, 2017, p. 6). “Vagueness, as a systemic property of languages, consists in the possibility of extending and restricting the boundaries of signs and therefore in the possible existence of non-categorical, but vague, fuzzy semantic boundaries” (*ibid.* p. 173). This concept of vagueness was posed by philosophers such as Russell (1923) and Wittgenstein (1953) and taken up by De Mauro (1982).

Although what we have seen above was given as a characteristic of partially linguistic or non-linguistic signs, it is instead precisely the characteristic of the linguistic sign. This is the extraordinary strength of linguistic signs, they are malleable, not discrete, variable, and because they are not inherently defined.

Even in the Peircian vision, icons and symbols fade into each other, or rather they are both features of the same linguistic sign that, depending on the context, and can show one side more than the other. However, they can never be encapsulated in categories strictly defined as self-excluding. Arbitrariness and even more so conventionality, are not the exclusive properties of “symbols” but belong to all linguistic signs, as well as to icons. The three grounds, Peirce emphasizes, are not in nature completely separate from each other: each phenomenon, in short, can be reported with prevalence to one or the other, but will probably exhibit characteristics of the other two as well.

**FIGURE 1:**
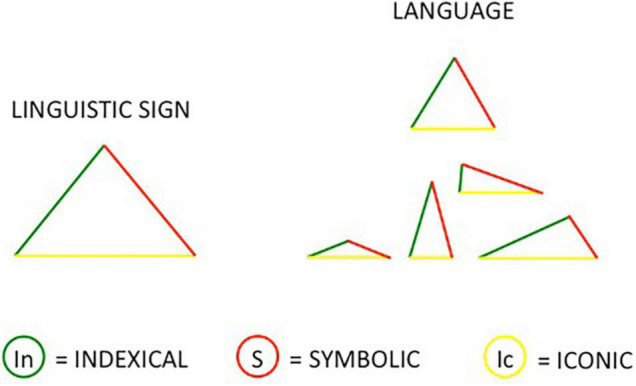
Three sides of the triangle: indexical, symbolic, and iconic.

We therefore arrive at a new representation of the three Peircian grounds or features that better represent their non-categorical dimension: not concentric circles (from the center to the periphery), not parallel lines that can activate together but also distinguish themselves, but three faces of the same triangle [as first proposed in Capirci (2018)]. In this perspective each linguistic sign is made up of three sides: indexical side, symbolic side, and iconic side. The key aspect is the proportional length of each side in building the triangle of linguistic signs. We can therefore have an equilateral triangle or other types of triangles depending on the length of each side (Fig 1).

**FIGURE 2:**
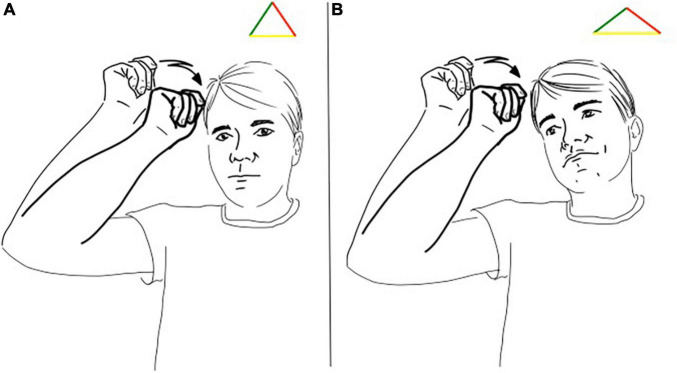
**(A,B)** Different triangles for the LIS sign “comb/combing”.

To illustrate the implication of this reasoning we can consider the LIS sign pictured in Figure 2A and glossed as “comb.” This sign is listed in LIS dictionaries and therefore has a conventional meaning and form, a symbol with a descriptive function. Nevertheless, if we look analytically at its realization, we can see that the hand is depicting a hand holding a comb and moving as if combing the hair, with a strong iconic feature. Finally, the location of the sign is the indexical feature pointing at the head.

So, in the case of a sign referring to “comb/combing,” we can see that all the triangle’s sides are simultaneously present and crucially, that the three sides of the triangle have the same length (see Figure 2A). In its citational form there is not a dominance of one side. The sign depicts the handling of the instrument, points to the effective location of the action and is highly conventional in the pair of form and meaning. However, in the signed utterances the sign can be used stretching one side, for example when enacting the event of combing the hair the “iconicity” side gets longer (see Figure 2B).

The lexicon of signed languages seem to be characterized by a high degree of iconicity and, at the same time, by the fact that the same signs may or may not appear iconic depending on the discursive and situational context. The same sign can vary these features while remaining the same. Signs can be used as both descriptions (lexemes or LU) and depictions (CAs or DCs, or TU) and should be considered as somewhere on a continuum between these two strategies rather than separating them in two distinct categories: “What is clearly symbolic at one level is part of an icon at another” (Armstrong, 1999, p. 146).

**FIGURE 3:**
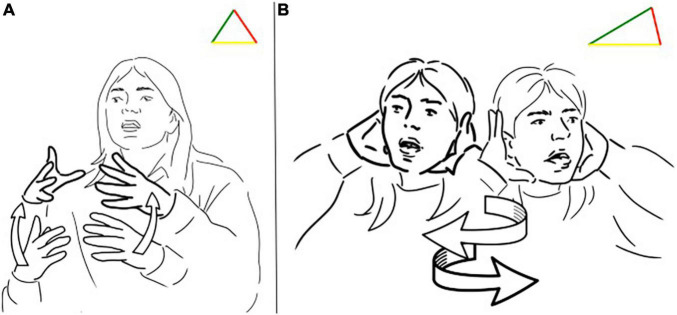
**(A,B)** Different triangles for the LIS sign “jar”.

The continuum is well illustrated in figure 3, which reports a part of the famous narrative retelling “Frog where are you?” In the signed utterance, the signer is at first introducing that there is a jar (Figure 3A). Then, the signer is enacting a dog being stuck in the jar, as shown in Figure 3B. In this case, we can observe that the signer first introduces a lexical unit translatable as “jar,” then she uses a transfer of person enacting the dog with her posture and non-manual components and simultaneously a transfer of form depicting the jar turned upside down. The handshape of the conventional sign is built on a transfer of form since it represents the circular shape of the jar (an equilateral triangle as in Figure 3A), therefore the iconic features of the conventional sign can be easily implied in a transfer construction, showing the fuzzy border of the distinction between lexical signs and transfer constructions. Also in this case, we can see that the iconic side of the triangle stretched to resemble the scene along with indexicality (see Figure 3B): the use of space is essential in providing information about the position of the jar being turned upside down and its locative relation with the dog.

**FIGURE 4:**
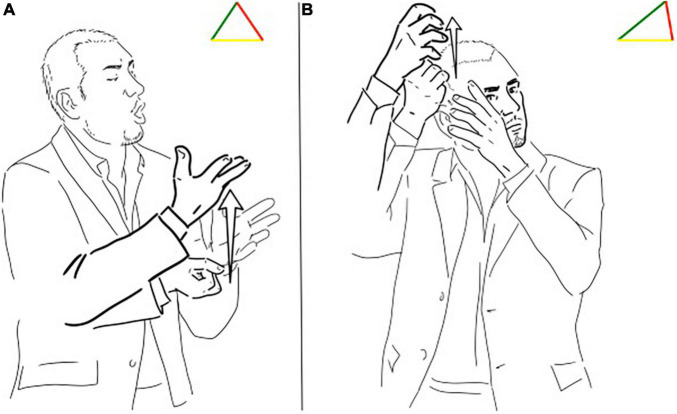
**(A,B)** Different triangles for the LIS sign “source”.

In a very different discourse type, a conference, we can observe a similar example: the same sign can present the dominance of a different feature in its use. A conference presenter at the beginning uses the sign translatable as “source” with an equal distribution of the three functions (symbolic, iconic, and indexical), represented in Figure 4A. Shortly after, the signer constructs the powerful metaphor of the mind as a source of ideas and thoughts moving the location of the sign in their head, stretching the indexical side of the triangle (Figure 4B). The distinction between lexemes and depiction often does not rely upon the sign itself but on its function in the signed utterance: the borders between what is a description or a depiction are fuzzy and determined by the signer use.

**FIGURE 5:**
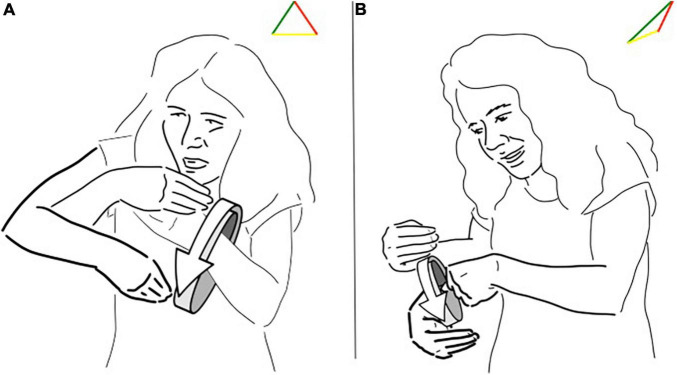
**(A,B)** Different triangles for the LIS sign “band/bandage”.

The signed utterance reported in Figure 5 belongs to a narrative about a horse which damages its leg crossing a fence and is nursed by a friendly cow. In the story, the same sign for “band/bandage” is used first as a lexical unit with a neutral value, like an equilateral triangle (Figure 5A). Then, the sign is used as a transfer, moving the sign in another location (and therefore stressing the indexicality features) to depict the action of the cow bandaging the horse’s leg (Figure 5B).

If we look at the level of the signed utterance, we can identify a dominance in one or two features: an iconic dominance, a symbolic or indexical one. Clearly, there is not a clear cut between what is a lexical unit and what is a transfer, each sign has a semiotic potential lying down in the three sides which can be exploited to stress one of its features.

**FIGURE 6:**
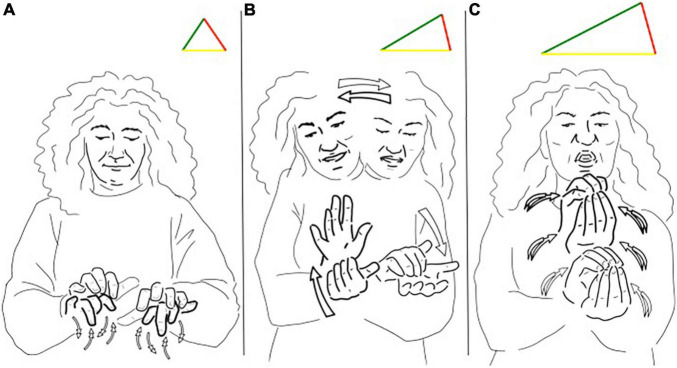
**(A–C)** Different triangles for the LIS sign “typing”.

Finally, we want to address the exploitation of the iconicity side in artistic contexts: the case of poems and theater. In signed poems, it is common to see how iconicity re-elaborates the meaning of signs, playing with the potential metaphorical and depictive power of the bodily articulators. The signs illustrated in Figure 6 are part of a poem about the deaf culture and the use of the old teletypewriter (TTY): a telecommunication device for the deaf largely used in Italy in the past years. The signer is describing the complex relationship between the user and the machine providing cold and frustrating communication. The sign referring to the act of typing on a keyboard (an equilateral triangle in Figure 6A) is reformulated to describe the act of writing/writing back (Figure 6B) and ending with the frustration of doing so (Figure 6C), stretching the iconic and indexical side of the triangles.

In this case, the semiotic resources of the sign presented in Figure 6A are poetically exploited to create a triadic unit with a greater iconic and indexical dominance in Figures 6B,C. The movement and expression of the signer depict the rate of the action and the emotional content related to it, while the location and orientation of the hands point to the reciprocal construction.

The visual modality allows for the use of space as well as multiple articulators (both hands, torso, head, face expression) for linguistic encoding, providing ample opportunity for exploitation of simultaneity in signed languages. Signers can employ iconicity to represent the information present in events as it is available in the real world and in order to encode actions and interactions. The signer can make use of the affordances of the visual modality by mapping the referent onto the signer’s body (e.g., through facial expression, eye gaze, and/or torso) and at the same time encoding the action by one of the hands.

Recently, the use of such iconic simultaneous constructions has been shown to increase with the increase of informative demands indicating that simultaneity can be used to achieve communicative efficiency by Slonimska et al. (2020, 2021). Simultaneity has profound consequences on the whole linguistic structure of signed languages. A semiotic structure is thus created in which it is not possible to distinguish, either at the level of a single unit or at utterance level, if this belongs to the category of symbol, icon, or index. We can find products simultaneously, a symbol with an icon, and/or an index. The overall structure of the sentence/utterance will have a greater or lesser degree of one of these elements, the signer will be describing, indicating, and depicting in more or less marked measures.

**FIGURE 7:**
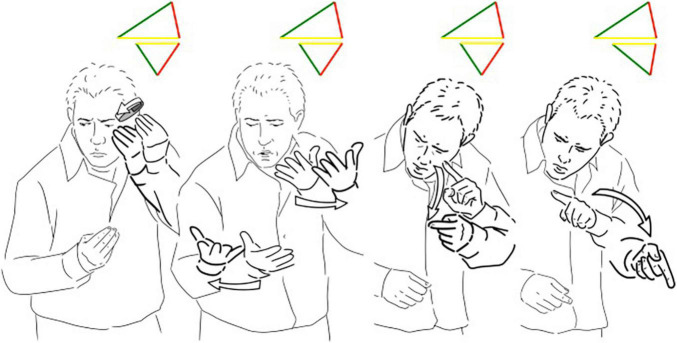
A brief utterance from the theater monolog in LIS.

In the case of a theater monolog, we can observe the complex interplay between different levels. Figure 7 illustrates a brief sentence from the monolog in which the signer talks about the everyday deaf experience and compares different types of pads to put under the armpit to absorb all the sweat coming from extensive signing. From the head movement, the facial expression and the direction of the eye gaze the signer depicts that he is talking to himself, he is performing a transfer of person. These signs are part of a complex transfer in which the non-manual articulators (eye gaze, oral components, and the head movements) and the manual articulators (right and left hand) can be considered as two different triangles (each one having its three features) presented simultaneously.

Therefore, in Figure 7 for each sign there are two triangles simultaneously displayed, one referring to the transfer of person mainly expressed by the “non-manual” components (the four upper triangles, with the iconic and indexical sides longer) and the other one referring to the lexical units performed with the hands (the four lower triangles). There is the level of performing the agent (who I am) and the level of telling (what I am signing).

### A Triangular Semiotic Model for Signers’ Head Movements

The first application of this triangular semiotic model was made by Puupponen (2019).

Puupponen presents a typology of head movements and their iconic, indexical, and symbolic features based on Peircean perspective and uses the visualization of triangles as presented by Capirci at the ISGS Conference in South Africa (2018).

The author argues that head movements present at the same time all three: iconicity, indexicality, and symbolic features, even though: “It may, however, be that these different strategies of signification emerge in different proportions in different head movement types” (Puupponen, 2019, p. 23). For example, the nodding/shaking head for affirmation/negation is a movement type showing a great proportion of indexical and symbolic features, while the iconic one is the smaller side of the triangle. On the contrary, the head movement following the time line metaphor has strong indexical and iconic features and a smaller symbolicity. Puupponen applied Capirci (2018) visualization to head movements and stressed that the symbolic side of the triangles, the red one as presented in Figure 1, is definitely not the more prominent one. The triangular model calls upon a perspective that include indexicality, iconicity and symbolicity as equals in language’s economy.

In conclusion, Puupponen rejects the distinction within non-manuals between categorial/grammatical non-manual and gradient/uncategorical non-manuals, declaring that in support of this distinction there is not enough empirical evidence and that indeed the results of some recent studies do not confirm this type of theoretical distinction (Puupponen et al., 2015).

## Conclusion

The social anthropologist Jack Goody (2000) in “The Power of the Written Tradition” (2000), a collection of nine essays, claims: “Words everywhere have meanings. But dictionaries do not only teach how to spell; they spell out meanings in a standardized way, ‘dictionary definitions,’ which then become the norm and the starting point of a discussion” (p. 144).

In this paper, we discussed how the need to hierarchically divide signs arose precisely from the need to establish which linguistic units are best entered in a dictionary (Johnston and Schembri, 1999) and by the need to code or annotate signed language data (Cormier et al., 2012).

Nevertheless, the discussion of the proposed models brought forth different questions: are there any standard signs (as opposed to others that are less or not standard at all) outside of dictionaries? Does the difference between standard/frozen/conventional/discrete lexicon and “productive”/little or non-conventional/gradient/unstable lexicon exist outside of dictionaries and our coding? Are the signers aware of using something fixed/conventional and something variable/unconventional in the flow of their communication?

Signed languages are “oral” languages, used in real face-to-face communication, without (to date at least) their own written form despite our efforts (by scholars, linguists) to “harness” them in written/discrete forms. Signs seem to fully respond to the description made by scholars of spoken communication, in which it is impossible to trace discretion and stability and where communication is in constant dynamic flow. “Oral tradition languages,” i.e., spoken languages lacking a written form, showing little or no codification, used exclusively for face-to-face communication, etc., are the languages that signed languages have most in common with.

We have discussed different models that tried to propose a solution to overcome a structuralist approach to signed language, applied at the very beginning of signed language research. Some models have been proven to be more effective than others, nevertheless, an even greater effort is needed by the field of signed language research to leave the patterns we have inherited, which lead us to categorize and divide into boxes (or circles or lines) elements that instead jump from one box to another.

As we have tried to show, it is not necessary to divide signs into symbols and icons, but rather it is more realistic to find symbolic iconic signs: “icons in which the likeness is aided by conventional rules” (Peirce C.P. 2.279). Iconicity cannot just be regarded as an accidental feature of the surface form of signs, we must instead acknowledge that it is a proper structural device (Pietrandrea, 2002; Russo, 2004), and a permanent feature in signs.

As argued by Boyes-Braem (1981), in signed languages the hands are used with a linguistic purpose. The hands are employed in daily life in many tasks, such as pointing, manipulating objects, counting, and representing objects. It makes economic sense that signed languages should make efficient use of this pre-codification of the hands in the creation of signs. There is no need to adjust a four-dimensional world to the linearity of the acoustic channel (Hockett, 1978). The peculiar nature of the articulators and the medium employed in signed languages play an important role in preserving iconicity.

Boyes-Braem’s argument can be easily extended to explain the linguistic use of the body. The speaker’s body is always present in signed language discourse. Again, it makes economic sense to exploit this presence to express meanings that are related to parts of the body (see also Borghi et al., 2014; Tomasuolo et al., 2020).

Since the beginning of modern signed languages studies, researchers have recognized the existence of two kinds of constituent elements. However, in most past and current research only one type of such elements has been granted the status of “truly linguistic items,” while the other one has been, and for the most part continues to be classified either as “non-linguistic, gestural, pantomimic items,” or as “partially linguistic,” but non-lexical.

On the contrary, we proposed to view each signed linguistic unit as a triadic union of iconic, symbolic, and indexical features, all immanent in the unit and potentially exploitable in signed discourse. Seeing each linguistic unit as a triangle helps the linguist to deconstruct the rigid language/gesture hierarchy, since it shows that conventionality coexists with iconicity and indexicality. Moreover, our discussion of signed utterances taken from different discourse types shows how the categories of “lexical unit” and “transfer unit” are actually fuzzy and context-dependent. In this respect, the effort provided by cognitive linguists in explaining the difference between core lexicon and classifiers should be followed by signed language research (Wilcox and Occhino, 2016; Lepic and Occhino, 2018; Lepic, 2019).

We have thus come to the realization that it is most adequate to view the linguistic unit as underspecified, deformable, not systematically discrete, placed in a dynamic flux, negotiable, and context-dependent. The meaning of words or signs is not fixed, given that the structure of language is characterized by a great plasticity that makes it possible to interpret it according to every different context (De Mauro, 1982, 1991, 2000). Signs and words (with gestures, ideophones, prosody) can be used both as descriptions and depictions, and we should look at usage events as objects constructed on and potentially used to express these three semiotic features, instead of separating them into three distinct categories.

## Data Availability Statement

The data analyzed in this study is subject to the following licenses/restrictions: The dataset is taken from video collections of theater performances, conferences and thesis studies. Consent forms do not support public access to video or media data. Requests to access the data should be directed to AdR, alessio.direnzo@istc.cnr.it.

## Author Contributions

OC conceptualized and design the study, conceived the “triangle semiotic model” and visualized it in the figure, and wrote the original draft of the entire manuscript. CB drafted one section and contributed substantially to the final writing of the manuscript. AdR transcribed and organized the video corpora and designed and edited all the figures. All the authors selected the data and wrote the descriptions of the data. All the authors thoroughly discussed and revised critically all the sections of the manuscript and approved the submitted version.

## Conflict of Interest

The authors declare that the research was conducted in the absence of any commercial or financial relationships that could be construed as a potential conflict of interest.

## Publisher’s Note

All claims expressed in this article are solely those of the authors and do not necessarily represent those of their affiliated organizations, or those of the publisher, the editors and the reviewers. Any product that may be evaluated in this article, or claim that may be made by its manufacturer, is not guaranteed or endorsed by the publisher.

## Footnotes

1 The complexity and overelaboration of signed phonology leaded Stokoe to propose in 1991 a completely different model: the semantic phonology.

2 According to Peirce “A sign, or representamen, is something which stands to somebody for something in some respect or capacity. It addresses somebody, that is, creates in the mind of that person an equivalent sign, or perhaps a more developed sign. That sign which it creates I call the interpretant of the first sign” (CP 2.228). The technical term “representamen” has a broader meaning than the term “sign,” as it can also refer to the first element of semiotic processes which do not have an interpreter with a mind and therefore cannot have an interpretant of a mental character.
